# Cross-subject affective analysis based on dynamic brain functional networks

**DOI:** 10.3389/fnhum.2025.1445763

**Published:** 2025-04-14

**Authors:** Lifeng You, Tianyu Zhong, Erheng He, Xuejie Liu, Qinghua Zhong

**Affiliations:** ^1^School of Physics, South China Normal University, Guangzhou, China; ^2^School of Social Sciences, Nanyang Technological University, Singapore, Singapore; ^3^School of Electronic Science and Engineering (School of Microelectronics), South China Normal University, Foshan, China

**Keywords:** EEG, emotion recognition, dynamic brain function network, subject independence, subject and trial independence

## Abstract

**Introduction:**

Emotion recognition is crucial in facilitating human-computer emotional interaction. To enhance the credibility and realism of emotion recognition, researchers have turned to physiological signals, particularly EEG signals, as they directly reflect cerebral cortex activity. However, due to inter-subject variability and non-smoothness of EEG signals, the generalization performance of models across subjects remains a challenge.

**Methods:**

In this study, we proposed a novel approach that combines time-frequency analysis and brain functional networks to construct dynamic brain functional networks using sliding time windows. This integration of time, frequency, and spatial domains helps to effectively capture features, reducing inter-individual differences, and improving model generalization performance. To construct brain functional networks, we employed mutual information to quantify the correlation between EEG channels and set appropriate thresholds. We then extracted three network attribute features—global efficiency, local efficiency, and local clustering coefficients—to achieve emotion classification based on dynamic brain network features.

**Results:**

The proposed method is evaluated on the DEAP dataset through subject-dependent (trial-independent), subject-independent, and subject- and trial-independent experiments along both valence and arousal dimensions. The results demonstrate that our dynamic brain functional network outperforms the static brain functional network in all three experimental cases. High classification accuracies of 90.89% and 91.17% in the valence and arousal dimensions, respectively, were achieved on the subject-independent experiments based on the dynamic brain function, leading to significant advancements in EEG-based emotion recognition. In addition, experiments with each brain region yielded that the left and right temporal lobes focused on processing individual private emotional information, whereas the remaining brain regions paid attention to processing basic emotional information.

## 1 Introduction

Emotion is a crucial mental state that significantly influences cognition, decision-making, and behavior, playing a pivotal role in people's daily lives. As a result, human emotion recognition has emerged as a focal point of research in various fields, such as cognitive science, computer science, and artificial intelligence (Guangming et al., 2021). Understanding the mechanisms of brain function related to emotions holds great significance for enhancing automatic emotion analysis, and human-computer interaction, as well as assisting in the detection and treatment of emotion-related brain diseases and psychological disorders.

Emotional intelligence has been in the process of being mapped out since the concept of “emotional computing” was introduced in 1997. Emotions, a unique and complex intrinsic property of human beings, are slowly emerging in the form of human-computer interaction through technological simulations and commercial reconfigurations of productivity. In affective computing, emotion recognition is the most fundamental and important element of research. Emotion recognition identifies various human emotional states mainly through the learning of non-physiological signals such as human facial expression, voice intonation, body posture, etc., and physiological signals such as EEG signals, ECG signals, EMG signals, etc. (Yuchi, 2017). Physiological signals are spontaneously generated under the direct control of the nervous system and the endocrine system and are less likely to be disguised and more realistic than non-physiological signals such as facial expressions and voice intonation (Ke et al., 2021). In recent years, studies in neuroscience and cognitive science have affirmed that the generation of emotion is closely linked to the activity of the cerebral cortex. Moreover, due to the ease of collecting EEG signals, their high temporal resolution, and the ability to directly record the activity of cortical potentials, EEG signals can reflect a person's emotional state more truly and reliably compared to other physiological signals. As a result, emotion recognition based on EEG signals has attracted widespread attention.

Emotion recognition using EEG signals typically involves four main steps: EEG signal acquisition, data preprocessing, feature extraction, and classification recognition (Zhongmin et al., 2022). Feature extraction is the key step in emotion recognition, and whether features highly relevant to emotion are extracted directly determines the performance of the recognition model. Commonly used EEG signal features include time domain, frequency domain, time-frequency, and nonlinear features (Yan, 2021). Wagh and Vasanth (2022) selected one electrode pair within each of the five brain regions to calculate various time domain features such as Hjorth parameter, kurtosis, skewness, etc., and extracted features such as power spectral density, energy, standard deviation, and variance by combining them with DWT, and classified the EEG signals using K Nearest Neighbor and Decision Tree. Chen et al. (2020) computed the L-Z complexity and wavelet detail coefficients of EEG signals and then performed Empirical Mode Decomposition and the average approximate entropy of the first four Intrinsic Mode Functions were used as features input into a LIBSVM classifier for dichotomous classification for emotion. Sarma and Barma (2021) utilized continuous wavelet transform and power spectral density to compute features using random matrix theory to distinguish EEG segments with high emotional content, automatically eliminating extraneous channels. However, the generation of emotional activity is the result of the interaction of multiple regions of the brain with each other. In addition, traditional features often neglect the spatial interaction information between brain regions, leading to a loss of important spatial information.

Graph neural network is a deep learning-based method for processing graph domain information, which can effectively explore the internal connections between nodes in graph-structured data. In recent years, there have been more and more researchers applying graph neural networks to the study of EEG emotion recognition, and trying to recognize and classify various types of emotions by combining time domain features (Huiyu, 2021). Noteworthy studies in this field include Bi et al. (2022)'s deep graph convolutional neural network (MdGCNN) with multi-domain fusion of feature inputs, which fuses differential entropy and Pearson correlation coefficient as network inputs, thereby eliminating the limitations of unimodal feature studies and extracting complementary high-level features. Zhang et al. (2021) improved the dynamic graph convolutional neural network (DGCNN) by introducing sparse constraints on the graph and solving the sparse constraint minimization problem, which ensures the convergence of the network model. Also, the experimental classification results are superior to the original model. The improved graph convolution model with dynamic channel selection designed by Lin et al. (2023) combines the advantages of one-dimensional convolution and graph convolution could capture both intra- and inter-channel EEG features. The spatio-temporal feature fusion convolutional graphical attention network model (STFCGAT) proposed by Li Z. et al. (2023) based on multichannel EEG signals utilizes a convolutional graphical attention network to fuse DE and FC features and extract high-level graph structural information for emotion recognition. Although similar studies mentioned above have begun to acknowledge the crucial role of inter-channel relationships in EEG, this structure has not been fully utilized to effectively learn significant EEG features. Moreover, most of the traditional features that are still extracted, such as DE, PSD, DASM, DCAU, etc., are then input into graphical neural networks for classification, but they do not describe the interactions and relationships between brain regions and channels better than the features of network attributes extracted by constructing the functional brain network.

Functional brain networks, which focus on whether brain regions and channels are functionally interconnected or separate from each other, are one of the main ways to characterize the synergistic work patterns among different brain regions and channels (Yuchi, 2017), reveal the functional relationships among different brain regions and channels, and are an effective way to reveal the interaction of cognitive information (Li C. et al., 2023). Zhang et al. (2023) calculated the phase lock values of different frequency bands of EEG signals, built a distance matrix, and put the distance matrix and its complexity into a machine learning classifier for classification. Li et al. (2022) computed the Pearson correlation coefficient, phase lock value and phase lag coefficient of the EEG signals, which were inputted into the proposed network model combining multiscale residual network (MSRN) and meta-transfer learning strategy (MTL) to alleviate the problem of large individual differences among subjects. For emotion recognition studies that employ brain networks, the construction of functional brain networks with static connectivity is mainly carried out. In other words, it is assumed that the connectivity of the brain network remains relatively stable within the time range of the computed EEG signals. However, it has been revealed that brain activity undergoes continuous changes at the level of seconds or even milliseconds as time elapses. Therefore, solely considering the static brain network will lead to the loss of some of the effective time-related features.

Existing investigations have achieved pleasing accuracy in subject-dependent emotion recognition. Furthermore, some researchers have also performed leave-one-subject-out (LOSO) validation experiments on the DEAP dataset. However, because of the non-stationary nature of EEG signals and the significant inter-subject variability, cross-subject emotion recognition often yields suboptimal outcomes. Bi et al. (2022)'s MdGCNN-TL, an extended network model of MdGCN, achieved an average classification accuracy of 65.89% for the LOSO validation experiments of triple classification on the DEAP dataset. Likewise, the sparse DGCNN approach presented by Zhang et al. (2021) achieved an average correct classification rate of 64.66% and 65.98% of the valence and arousal dimensions, respectively. Li et al. (2022)'s emotion recognition method based on MSRN and MTL achieved an average classification accuracy of 71.29% and 71.92% on the valence and arousal dimensions, respectively. The cross-subject emotional EEG classification model introduced by Quan et al. (2023) based on multi-source domain selection and subdomain adaptation, achieved average classification rates of 81.19% and 79.59% on the valence and arousal dimensions, respectively. The subject-independent LOSO validation results from the above studies on the DEAP dataset indicate that there is room for improvement in achieving satisfactory classification accuracy.

**Figure 1:**
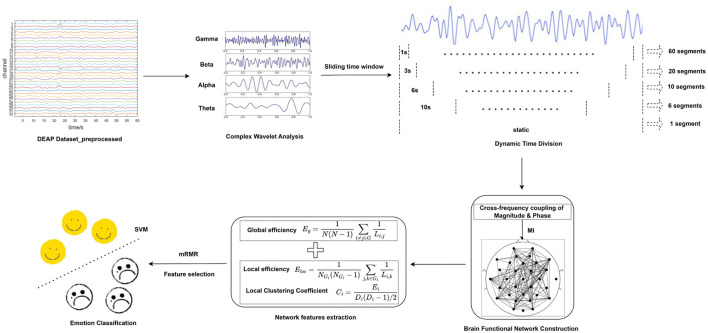
Flow chart of dynamic brain function network based emotion recognition.

To address these challenges, this paper proposes a novel approach that integrates time-frequency analysis and brain functional network analysis to construct dynamic brain functional networks across different frequency bands. This paper's main contributions are illustrated as follows: (1) Comparing the ability of static and dynamic functional brain networks to capture brain activity, i.e., brain network connectivity, it is concluded that high-precision dynamic functional brain networks are better able to capture more comprehensive and extensive connectivity between different regions of the brain over time. (2) The combination of time-frequency analysis and dynamic brain functional networks effectively integrates features in the time, frequency and spatial domains, reduces inter-subject variability, improves cross-subject emotion recognition performance, and provides strong support for the generalization ability of affective computing technology in practical applications. (3) The impact of various brain regions on emotion recognition was investigated. Following subject-dependent and subject-independent experiments, it was determined that the temporal lobe predominantly mediates the personalized processing of emotional pleasure levels, whereas other brain regions primarily engage in processing fundamental, common information during the processing of emotional stimuli. (4) In contrast to deep learning models, the method introduced in this paper offers a more intuitive insight into emotion recognition and brain function research, adopting a more explainable perspective. Figure 1 depicts the flowchart of emotion recognition based on dynamic brain functional networks, including dynamic segmentation of EEG signals, brain functional network construction and network attribute feature extraction and selection, and emotion classification.

## 2 Materials and methods

### 2.1 Dataset

**Figure 2:**
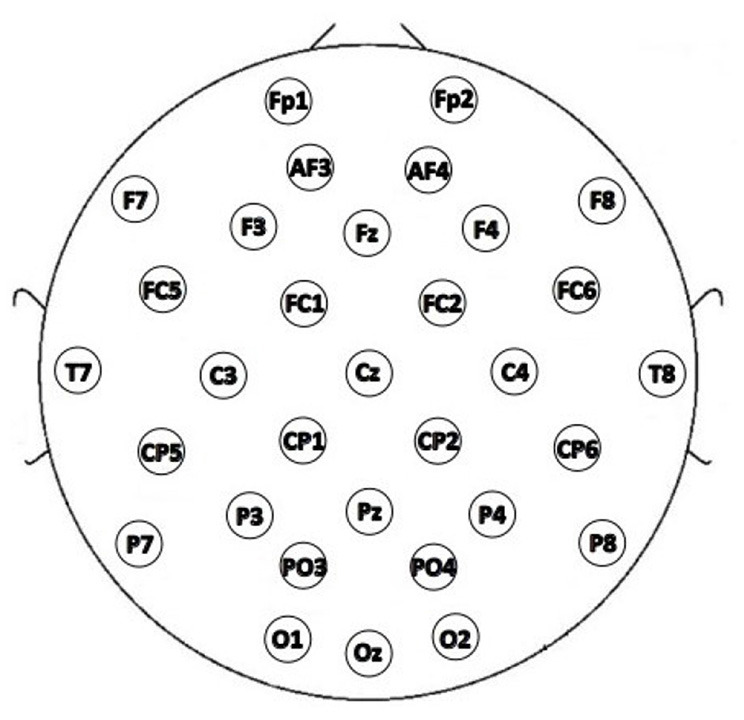
32-lead EEG channel distribution of DEAP dataset.

The data used in this paper were obtained from the DEAP dataset (Koelstra et al., 2011). The dataset recorded 40-lead data, which encompassed 32-lead EEG signals and an additional 8-lead physiological signals, from 32 healthy subjects (16 males and 16 females, with an age range of 19–37 years old, a mean age of 26.9 years old, and all right-handed). The 32-lead EEG channels are depicted in Figure 2. Each subject performed 40 experiments, i.e., watched 40 60-second music video clips of different types to evoke and record the physiological signals related to emotions, and assessed their emotional state in the range of 1–9 at the end of each experiment, based on the valence, arousal and other dimensions of the music video clips. The database provides two types of EEG data: one is the raw unprocessed data with a sampling rate of 512 Hz, and the other is the pre-processed EEG data (128 Hz downsampling, 4–45 Hz band-pass filtering, and removal of oculo-electrical disturbances, etc.), which is the latter chosen in the experiments of this paper.

The initial size of the data matrix for each subject was 40 trials × 40 channels × 8,064 time points (corresponding to 63 seconds × 128 Hz). This matrix contained additional 8-conductor non-EEG signals as well as the baseline data from the first 3 seconds. To preprocess the EEG signals, the average of this baseline data was subtracted from the last 60 seconds of the signal to eliminate the influence of baseline signals. The 8-conductor non-EEG signals were directly removed, resulting in the final EEG signals being ready for further processing. The size of the preprocessed data matrix for each subject was 40 trials × 32 channels × 7,680 time points (corresponding to 60 seconds × 128 Hz). Regarding the label data, a threshold value of 5 was set to classify the valence and arousal ranges into high and low categories, respectively (score of 5 was classified as high). This categorization likely simplifies the classification task and allows for a binary emotion classification approach. For the valence, data labels with High Valence (HV) and Low Valence (LV) were set to 1 and 0, respectively. Similarly, for the arousal, data labels with High Arousal (HA) and Low Arousal (LA) were set to 1 and 0, respectively. The combination of valence and arousal can be used to construct the four-classification labels for EEG emotion recognition, i.e., HVHA, HVLA, LVHA, and LVLA. The dimension sizes of the final EEG data and labels are shown in Table 1.

**Table 1:** Composition of the processed DEAP dataset.

	**Size**	**Details**
Data	32 × 40 × 32 × 7,680	Subject × video/trial × channel × data (60 sec.)
Labels	32 × 40 × 3	Subject × video/trial × label [valence, arousal, valence & arousal (four-class)]

### 2.2 Functional brain network analysis

#### 2.2.1 Complex wavelet analysis

**Figure 3:**
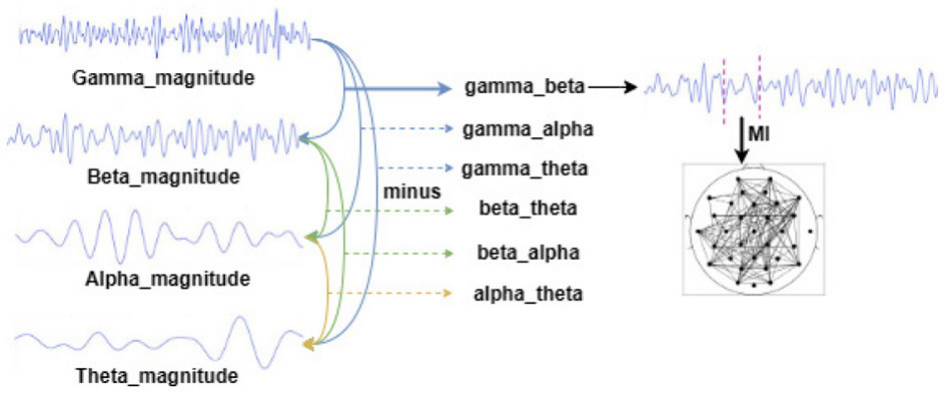
Schematic diagram of constructing a functional brain network based on the amplitude difference between frequency bands (constructing a functional brain network based on the phase difference between the frequency bands is the same as that).

For the above pre-processed DEAP dataset, the EEG signals in different frequency bands of each subject were analyzed by complex Morlet wavelet analysis, and the frequency bands were 4–8 Hz, 8–12 Hz, 12–32 Hz, and 32–42 Hz, corresponding to theta, alpha, beta, and gamma rhythms. Then, we extracted the amplitude and phase information of each rhythm and took the difference between the amplitude and phase of each rhythm to prepare for the subsequent construction of the functional brain network. In this way, the amplitude and phase based on the four frequency bands can be obtained from six copies of the difference data, respectively. Figure 3 constructs a flowchart for constructing a functional brain network based on the amplitude difference between the frequency bands.

#### 2.2.2 Dynamic time division

Functional brain networks are divided into two categories: static and dynamic connections. A static brain functional network is one in which the brain activity i.e., the functional topology of the network is kept relatively stable by default for long time periods of acquired EEG signals. It has been shown that brain activity varies continuously over time at the second or even millisecond level. Dynamic brain function networks solve the problem of time-varying information loss in static function networks, which usually use sliding time windows to divide a given EEG signal into small time segments, i.e., dynamic brain function networks are composed of multiple static brain function networks.

To determine if dynamic time division is beneficial for the brain function network in capturing temporal information, the performance of the static brain function network (without a sliding time window or with a 60-second sliding time window) was compared with that of the dynamic brain function network using different sliding time window lengths for emotion classification. Since brain activity, or the brain function network, changes over time at the level of seconds or even milliseconds, a fine division of the time dimension of the EEG signal can capture more information. Therefore, the sliding time window length was set to gradually change from coarse-grained to fine-grained time division. Corresponding to this, the sliding time window length of the dynamic brain function network in the experiments of this paper was set to 10s, 6s, 3s, and 1s step by step. When the static brain functional network is employed, the 60-second signal is regarded as 1 segment for further processing.

#### 2.2.3 Brain functional networks construction based on MI

The size of the network functional connectivity is generally estimated by the correlation, coherence, mutual information, phase lock value and phase lag coefficient between channels, i.e., nodes, in different regions of the EEG, followed by an artificial selection of thresholds to determine the existence of edges between the channels to complete the construction of the functional network. Considering the nonlinearity of EEG signals, in this paper, mutual information is chosen as a measure of functional connectivity between electrodes.

Mutual Information (*MI*) is a commonly used information measure in information theory to evaluate the degree of dependence between two random variables, representing the amount of information contained in one random variable about another random variable, and in the field of EEG emotion recognition, *MI* can also be used to estimate the magnitude of synchronization between EEG signals in two electrodes. The *MI* between two random variables can be defined as:


(1) $$\begin{array}{l} MI_{xy}=H_{x}+H_{y}-H_{xy} \end{array}$$

where $H_{x}=-\overset{M}{\underset{i=1}{\sum }}p_{i}lnp_{i}$ represents the information entropy of variable *x*, *H*_*y*_ is the same, *p*_*i*_ represents the probability density, $H_{xy}=-\overset{M}{\underset{i,j}{\sum }}p_{i,j}lnp_{ij}$ represents the joint probability information entropy between *x* and *y*, and *p*_*ij*_ represents the joint probability density between the variables. The greater the value of *MI* between two variables, the greater the synchronization between the EEG signals of the two electrodes is represented.

Based on the aforementioned preliminary processing of EEG signals, by setting the window to 60, we can obtain a data size of 32 channels × 12 for each subject per trial. That is, without dividing the signals, a 32 × 32 channels × 12 functional connectivity matrix is generated by sequentially calculating the synchronization between different channels based on *MI*. Due to significant inter-individual differences, it is not feasible to binarize the functional connectivity matrices of all subjects using a uniform threshold. Therefore, the elements within each functional connectivity matrix are sorted in ascending order to obtain a 1 × 1024 data matrix, and the data at position 0.3 (i.e., data located at position 307) is taken as the threshold. If the data is less than the threshold, it is set to 0, and the connecting edge is ignored; if it is greater than the threshold, it is set to 1, and the connecting edge is retained. Subsequently, the brain functional networks were constructed based on the binarization matrix. The number of networks constructed by the static brain functional network analysis and the dynamic brain functional network analysis under different sliding time windows was proportional to the number of time segments, e.g., the number of networks constructed by static brain functional network analysis was 12, and the number of networks constructed by dynamic brain functional network analysis under the 6-second sliding time window was 120 (12 × 10, the number 10 means that the 60-second signal was divided into 10 segments by the 6-second sliding time window).

#### 2.2.4 Network topology attribute features extraction

After completing the abstraction of the network, the graph theory is used to extract the network related attributes. Functional integration and functional segregation of brain networks are two basic principles of the operation of complex brain networks that can quantify the global and local information processing capabilities of the brain. Functional integration refers to the brain's ability to integrate information from different regions, i.e., the ability to process global information, emphasizing the interactive relationship between multiple brain regions. Global information processing capacity is typically measured by two network properties, global efficiency and average shortest path length. Functional segregation refers to the ability of the brain to process specific information within regions, i.e., the ability to process local information. The ability to process local information is usually measured by network properties such as local efficiency, clustering coefficient, and node degree. Functional integration and functional segregation in brain activity are interdependent and complementary. When performing an activity task, it is necessary for each brain region to simultaneously invoke multiple brain regions for mutual coordination and integration, while maintaining their respective functional specificities (Tononi et al., 1994).

Based on the metrics of brain functional integration and functional separation, we compute three network attribute features, namely the global efficiency, local efficiency, and local clustering coefficient. This is because the shortest path length requires the network to be fully connected to be meaningful. However, the choice of a threshold for functional network connectivity may lead to a network that is not fully connected. Therefore, we decide to discard this feature.

Characteristic Path Length *L*_*p*_ is the average of the shortest paths from any node *i* in the network to other nodes within the network *G*. It is a key parameter for studying the complexity of brain networks, and can reflect the information transfer ability of brain regions. The smaller the characteristic path length,the faster the information transfer speed between networks. The definition of characteristic path length is shown below:


(2) $$\begin{array}{l} L_{p}=\frac{1}{N\left(N-1\right)}\underset{i\neq j\in G}{\sum }L_{i,j} \end{array}$$

where *L*_*i, j*_ denotes the shortest path between node *i* and node *j*, and *N* is the number of network nodes.

The characteristic path length is practical only for connected nodes. If there is no path connection between two nodes, the shortest path length obtained will be infinite. In order to overcome this limitation, the efficiency between the nodes is defined to be the inverse of the shortest path 1/*L*_*i, j*_ and the Global Efficiency *E*_*g*_ is defined as shown below:


(3) $$\begin{array}{l} E_{g}=\frac{1}{N\left(N-1\right)}\underset{i\neq j\in G}{\sum }\frac{1}{L_{i,j}} \end{array}$$

Local Efficiency *E*_*loc*_ is used to measure the degree of local network aggregation formed by network node *i* and its neighboring nodes. The local efficiency of node *i* is defined as shown below:


(4) $$\begin{array}{l} E_{loc}\left(i\right)=\frac{1}{N_{G_{i}}\left(N_{G_{i}}-1\right)}\underset{j,k\in G_{i}}{\sum }\frac{1}{L_{i,k}} \end{array}$$

where *G*_*i*_ is the subgraph formed by node *i* and its neighboring nodes, *N*_*G*_*i*__ denotes the number of nodes in subgraph *G*_*i*_, *j* and *k* are nodes in *G*_*i*_ that are different from each other, and *L*_*i, k*_ is the shortest path length between *i* and *k*.

The clustering coefficient is an important parameter for measuring the degree of aggregation of a network, reflecting the degree of connectivity between neurons in the local functional regions of a brain network. The local clustering coefficient *C*_*i*_ of node *i* is defined as the ratio of the number of edges directly connected to other nodes in the network to the maximum possible number of edges connected to other nodes in the network, as follows:


(5) $$\begin{array}{l} C_{i}=\frac{E_{i}}{D_{i}\left(D_{i}-1\right)/2} \end{array}$$

where *D*_*i*_ denotes the number of neighboring nodes of node *i*, and *D*_*i*_(*D*_*i*_ − 1)/2 denotes the maximum number of possible edges between *D*_*i*_ nodes.

The above network attributes are obtained in turn for the generated brain function networks, for each constructed brain functional network, global efficiency (1 × 1), local efficiency (1 × 32), and local clustering coefficient (1 × 32) were calculated separately, and the number of features of each brain functional network was obtained to be 1 × 65, which can be analogized to obtain the number of features calculated by constructing a brain functional network for each subject under each trial as (12 × 60/t × 65), t is the length of the sliding time window. The above feature extraction is partially implemented on matlab R2018b.

### 2.3 Emotion classification

In this paper, two types of classification tasks are conducted. The first is the binary classification of valence and arousal separately, and the second is the four-class classification of the four quadrants of the valence-arousal plane.

In terms of data balance, the composition of binary labels for valence and arousal, as well as four-class labels, was assessed across 32 subjects. On average, HV occurred 22.63 times (56.58% of 40) per subject across 40 trials. The mean occurrence of HA was 23.56 times (58% of 40). The average occurrences of HVHA, HVLA, LVHA, and LVLA were 8.13, 9.25, 8.31, and 14.31, respectively. These results indicate that the data distribution is relatively balanced, as the mean occurrence of each class is equitable.

Owing to the large number of calculated features, there exist redundant features which need to be pruned. In this paper, we opt to use Max-Relevance and Min-Redundancy (mRMR) for feature selection. This paper focuses on exploring the acquisition of temporal information by dynamic brain function networks, and emotion classification methods are not the main focus of the study. Therefore, SVM, which has better classification performance and is widely used, is chosen for classification. Based on the balance of different labeled data, this paper chooses accuracy as an evaluation metric. In order to improve the generalization performance of the classifier, the training process uses repeated hierarchical 5-fold cross-validation combined with the grid search method to find the optimal parameters of kernel function and C. After training the classifier on the training set to obtain the optimal parameters of the model, the trained model predicts the probability of the test set belonging to each category, and this process is repeated 100 times. Finally, the category to which it ultimately belongs is determined based on the average of the 100 prediction probabilities. In order to evaluate whether the shortening of the sliding time window led to a significant improvement in classification accuracy, a Wilcoxon signed-rank test was conducted on the paired data for each experimental condition. The test was performed in the two-tailed case, and the significance level was set at 0.05. All of the above processes are implemented on python 3.7.0, where the version of scikit-learn used is 1.0.2.

### 2.4 Experimental setup

In this paper, subject-dependent, subject-independent, and subject- and trial-independent experiments were conducted to gradually explore the prediction of the model in the cross-trial, cross-subject, cross-subject, and -trial cases.

**Figure 4:**
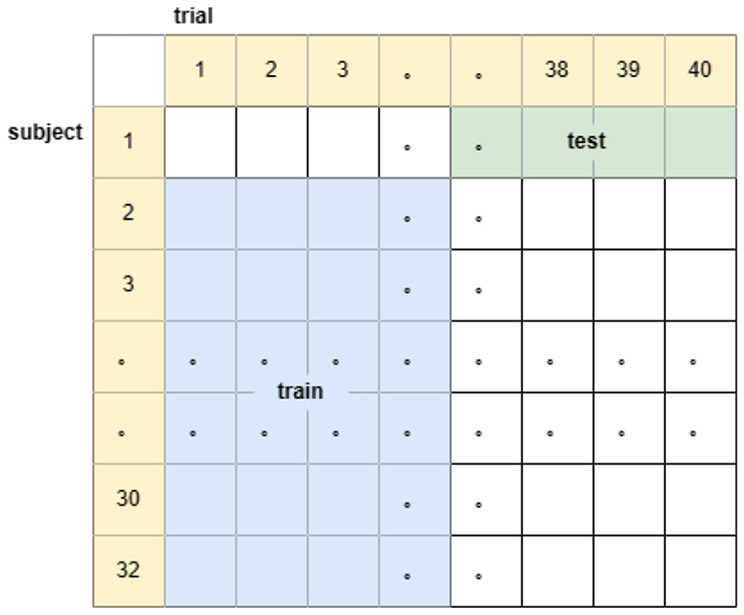
Schematic diagram of the division of the data set for subject, trial independent experiments.

(1) The subject-dependent experiment involves dividing the data of each subject (40 trials) into training and test sets (train:test = 3:1) respectively. Then, the training is looped 32 times to obtain the classification accuracy of the 32 subjects under the training model based on their own data from different trials. This corresponds to real-life scenarios where an individual's emotional response to a specific stimulus is known and used to predict their emotional response to other stimuli.

(2) The subject-independent experiments, i.e., leave-one-subject-out (LOSO) validation experiments, are sequentially selecting 1 subject as the test set and the remaining 31 subjects as the training set, cycling through the training 32 times to obtain the classification accuracy of the 32 subjects under the training model based on the data of other subjects. Corresponding to the real-life known emotional responses of some people to a given stimulus to predict the emotional responses of others to that given stimulus.

(3) The subject, trial independent experiment is to sequentially select one subject, randomly choose 20 of his 40 trial data as the test set, and the remaining 31 subjects choose the remaining 20 trial data as the training set (the dataset is divided in the way as in Figure 4), and cycle the training for 32 times to get the classification accuracy of the 32 subjects based on the training model of the other subjects with different trial data. Corresponding to the real-life known emotional responses of some people to given stimuli to predict the emotional responses of others to other stimuli.

## 3 Experimental results

### 3.1 Comparison of functional brain network maps under different sliding time windows

**Figure 5:**
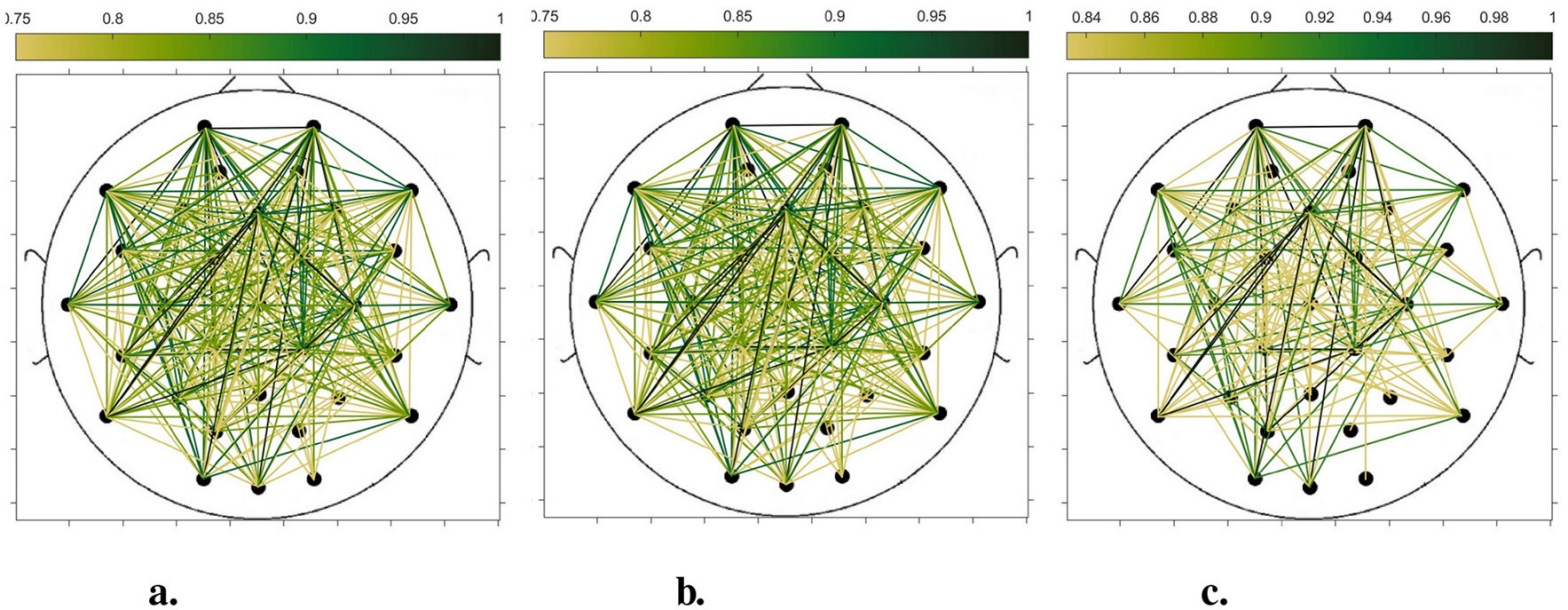
Brain functional network maps with retention of edges greater than 70%, 75%, and 80% of the maximum connectivity edge in the static condition. **(a)** sta-0.7. **(b)** sta-0.75. **(c)** sta-0.8.

**Figure 6:**
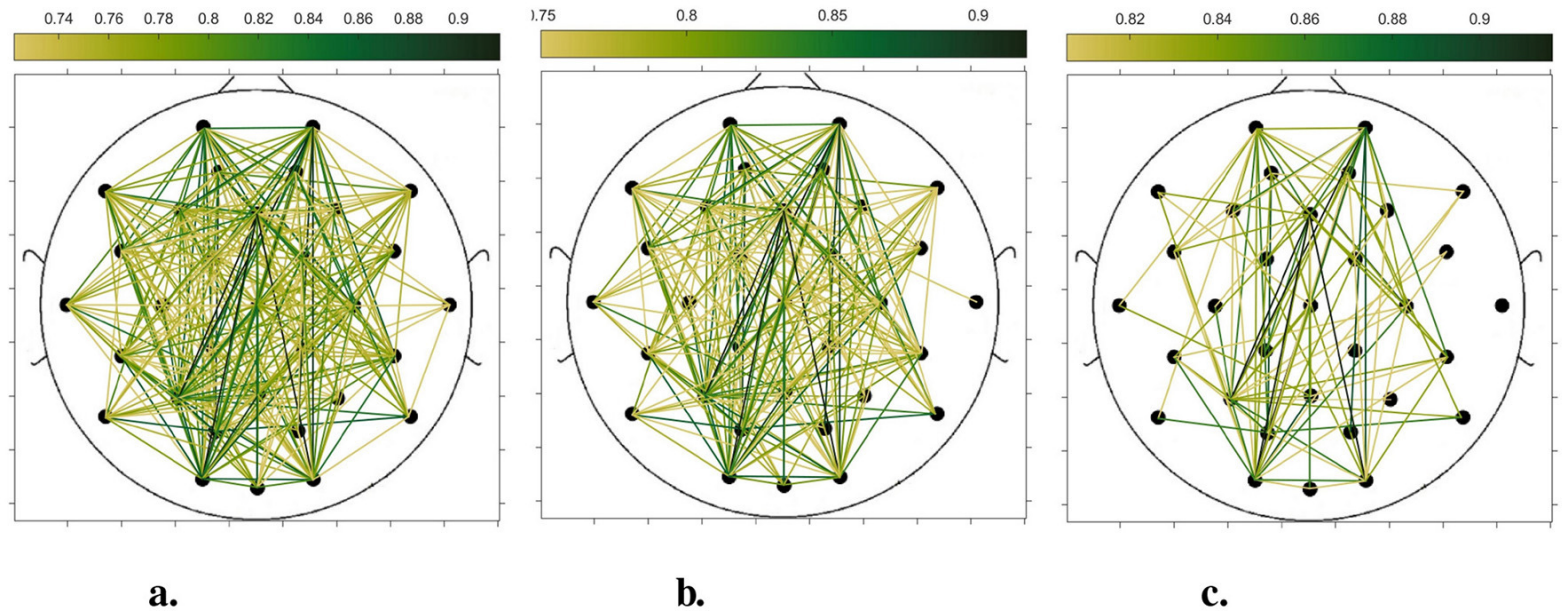
Brain functional network maps with retention of edges greater than 70%, 75%, and 80% of the maximum connectivity edge under a 10-second sliding time window. **(a)** 10-0.7. **(b)** 10-0.75. **(c)** 10-0.8.

**Figure 7:**
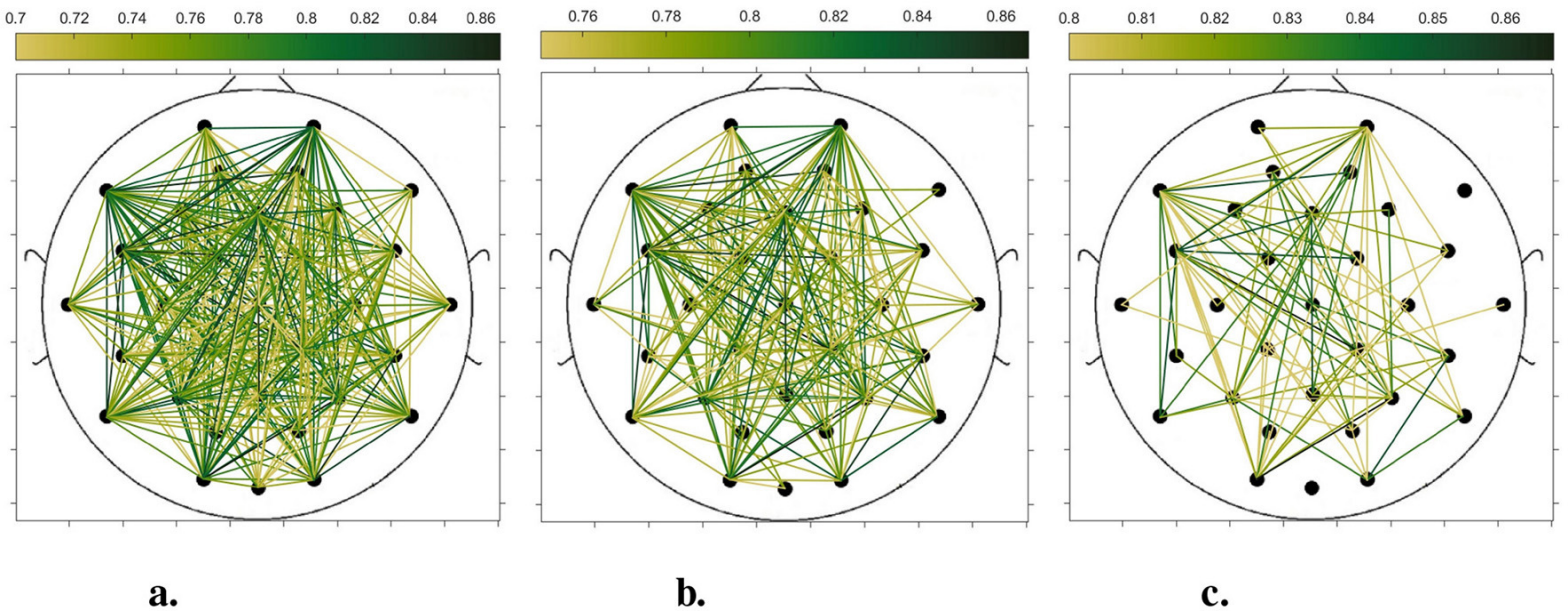
Brain functional network graphs for retaining edges larger than 70%, 75%, and 80% of maximal connectivity edges under a 6-second sliding time window. **(a)** 6-0.7. **(b)** 6-0.75. **(c)** 6-0.8.

**Figure 8:**
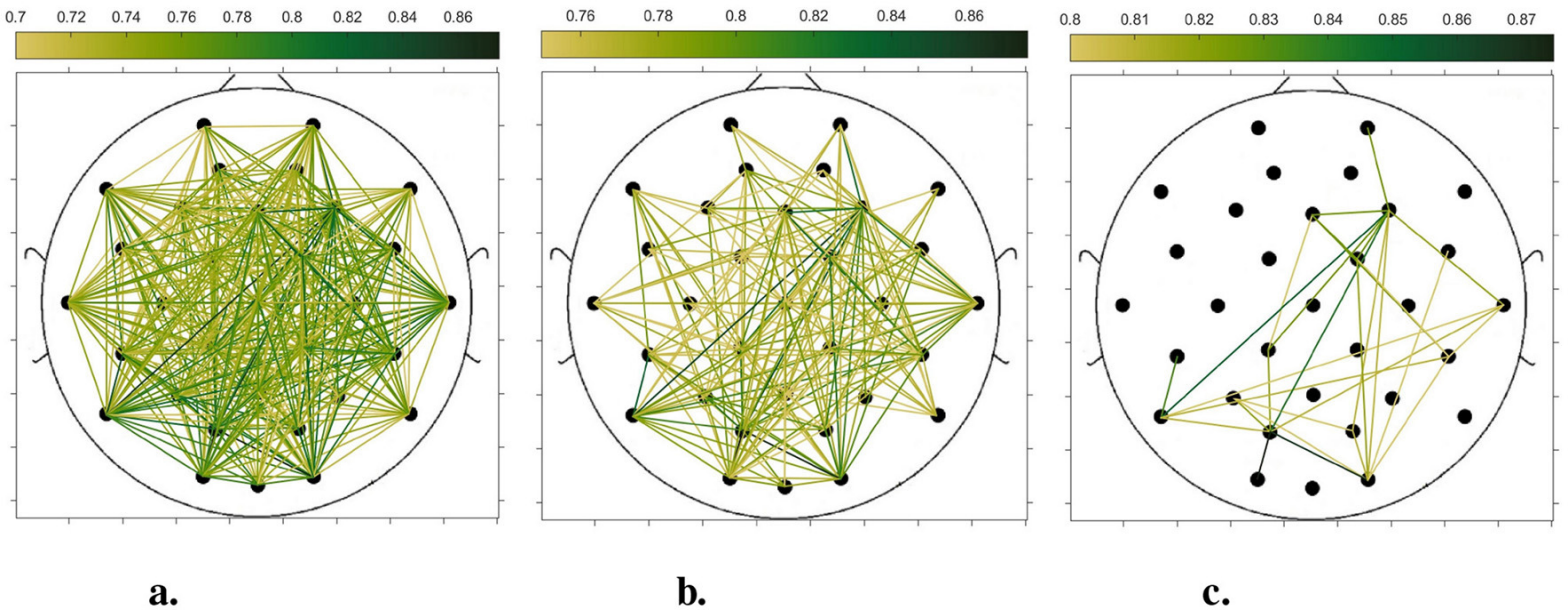
Brain functional network graphs for retaining edges larger than 70%, 75%, and 80% of maximal connectivity edges under a 3-second sliding time window. **(a)** 3-0.7. **(b)** 3-0.75. **(c)** 3-0.8.

**Figure 9:**
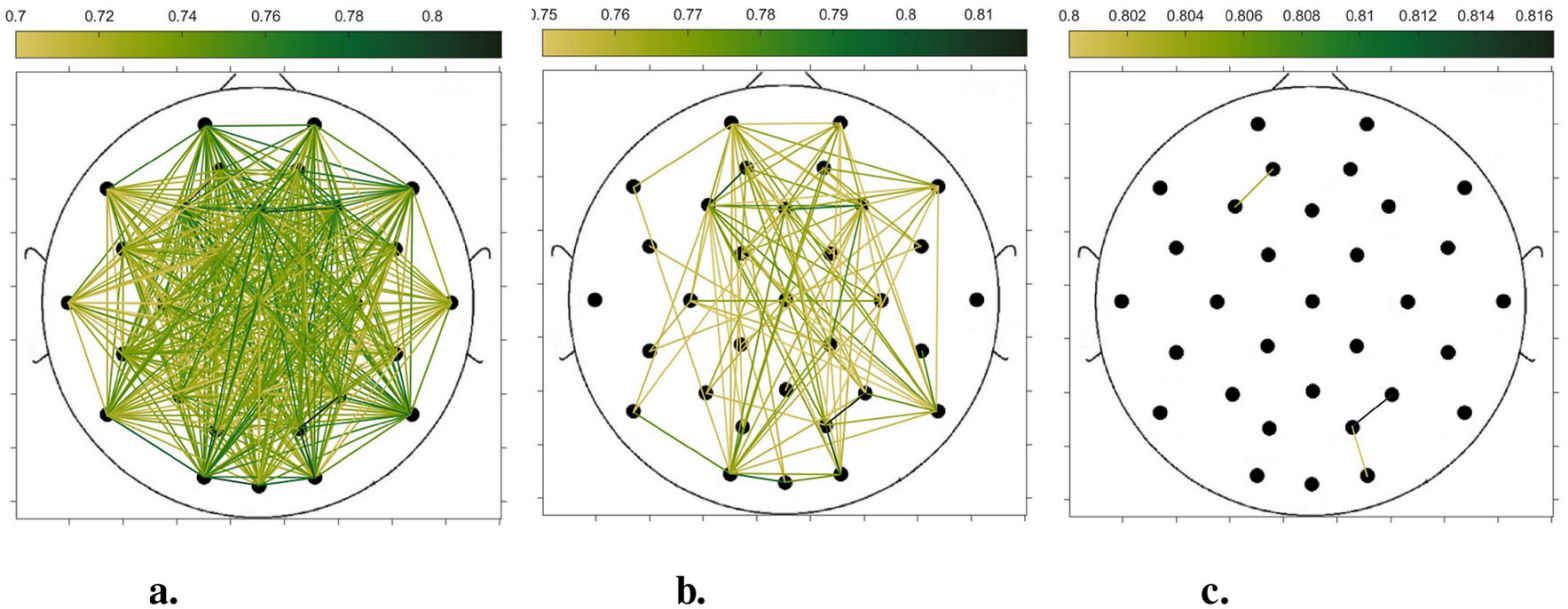
Brain functional network graphs for retaining edges larger than 70%, 75%, and 80% of maximal connectivity edges under a 1-second sliding time window. **(a)** 1-0.7. **(b)** 1-0.75. **(c)** 1–0.8.

In order to compare the connectivity between different channels of the static brain functional network and the dynamic brain functional network under different sliding time windows, all the edges in the constructed static brain functional network and the dynamic brain functional network were connected to form a larger and denser brain functional network respectively. Then according to the possible maximum connectivity of the network (e.g., the maximal connectivity of the static brain functional network was 12, and the maximal connectivity of the dynamic brain functional network under the sliding time window of 6 seconds was 120), we selected the edges with the number of connected edges greater than 70%, 75%, and 80% of the maximal connected edges, and drew the corresponding brain functional network graphs. The subject 1 trial 1 was selected for the demonstration to visualize the changes in the network connection information captured by the constructed brain functional network as the length of the sliding time window shortened. Figures 5–9 list static and dynamic brain functional network diagrams for subject 1 trial 1, where t in t-p denotes the dynamic brain functional network constructed under a sliding time window of t seconds, and p denotes the retention of edges greater than p times the maximum number of connected edges, and sta-p is the constructed static brain functional network.

Taking the comparison of the static brain functional networks and dynamic brain functional network diagrams that retain p times the number of edges based on their respective maximum number of connected edges into consideration, it can be found that when brain network connections composed of edges theoretically larger than 70% of the maximal connectivity edges are retained, as in Figures 5a, 6a, 7a, 8a, 9a, it is intuitively found that the density of brain network connections is progressively greater from the static brain network graphs to the dynamic brain network graphs with progressively shorter lengths of the sliding time window, indicating that the dynamic brain network is better able to capture a more comprehensive and broader range of connections among different channels. However, when brain functional network graphs consisting of edges larger than 75% and 80% of the respective theoretical connecting edges are retained, as shown in Figures 5b, c, 6b, c, 7b, c, 8b, c, 9b, c respectively. In this condition, the density of the brain functional network connection gradually decreases from the static brain functional network to the dynamic brain functional network with a gradually shortening sliding time window length. Considering the dynamic brain functional network constructs a larger number of brain functional networks compared to the static brain functional network, if the ability of the dynamic brain network to capture network connection information is the same as that of the static brain network, the network sparsity of each dynamic brain network graph should be greater than that of the static brain network graph, but the results show the opposite. Comparing the static functional brain networks with different numbers of edges, the network sparsity density undergoes the least amount of change. This indicates that the constructed static functional brain networks capture more repetitive information regarding edge connections (the global stability of the brain network connections) compared to the dynamic functional brain networks. This shows that the dynamic brain network has the ability to capture more functional connections over time than the static brain network. As the sliding time window is shortened, the dynamic brain network graphs with smaller time windows show less network density, which indicates that the higher the precision of the division, the more the dynamic brain network can capture functional connections based on a higher temporal resolution. This is consistent with the results shown by the functional brain network constructed by retaining more than 70% of the edges of the maximum connectivity edge, because the dynamic functional brain network can capture more temporally detailed functional brain network connections, and the production and change of emotion is accompanied by the change of the functional brain network connection structure. As a consequence, a denser functional brain network than the static functional brain network is captured when retaining more than 70% of the edges of the maximum connectivity edge.

### 3.2 Comparison of emotion recognition in static and dynamic functional brain networks

#### 3.2.1 Subject-dependent experimental results

Table 2 shows a comparison of the results of subject-dependent experiments averaged over 32 subjects based on static and dynamic functional brain networks (in the table Static represents the static functional brain network and Dynamic-t represents the dynamic functional brain network with a sliding time window of t). For the binary classification of valence and arousal dimensions, from the table, it can be seen that the average classification performance of the dynamically divided time-constructed brain function network is above 94.69%, which is greater than the highest classification performance of 90.31% under the static brain function network. The best classification performance under the arousal dimension is 100% when the length of the sliding time window is 3 seconds, and the best classification performance under the valence dimension is 99.69% when the sliding time window is 6 seconds. When each brain function network is used for the four-class classifications of emotion in terms of valence and arousal, a finer division of emotion is required. At this time, the dynamic brain function network constructed with a 1-second sliding time window achieves the best classification performance of 88.28%. This indicates that the dynamic brain function network can effectively capture the feature information of individual subjects.

**Table 2:** Accuracy of emotion analysis under subject dependence (mean ± std).

**Accuracy**	**Subject_dependent**		
	**Arousal(**%**)**	**Valence(**%**)**	**Valence** & **Arousal(**%**)**
Static	90.31 ± 10.15	88.75 ± 15.76	58.28 ± 21.67
Dynamic-10	94.69 ± 10.3	96.88 ± 8.08	70.69 ± 24.2
Dynamic-6	98.75 ± 3.31	**99.69** **±1.74**	79.31 ± 17.8
Dynamic-3	**100** **±0**	98.44 ± 8.7	82.76 ± 21.16
Dynamic-1	93.75 ± 16.54	95.31 ± 14.57	**88.28** **±19.31**

Bolded values are the best results obtained in different experiments.

The Wilcoxon signed-rank test was conducted on 32 subjects across three categorical scenarios. In the arousal dimension, no significant improvement in accuracy was observed for “Dynamic-1” compared to “Dynamic-6,” “Dynamic-10,” or “Dynamic-3” compared to “Dynamic-6.” However, in all other instances, there was a significant increase in accuracy when the sliding time window length was set to a shorter duration relative to a longer duration. For the valence dimension, the accuracy of sliding time windows with various lengths was significantly higher than that of the “Static” condition. In the context of four-class classification, no significant improvement in accuracy was noted for “Dynamic-1” compared to “Dynamic-3” or “Dynamic-3” compared to “Dynamic-6.” Nevertheless, in the remaining cases, a significant increase in accuracy was again observed when the sliding time window length was set shorter as opposed to being set longer.

#### 3.2.2 Subject-independent experimental results

For the classification of subject independence, i.e., leave-one-subject-out (LOSO) validation, as shown in Table 3, the results of the LOSO validation for 32 subjects were averaged to obtain the following results. Because of the reason of the large gap between subjects, it can be seen that the results are not as good as the subject-dependent results. However, because the individualization of subject-dependent experiments will be more prominent, the fluctuations between individuals will be larger, whereas with subject-independent experiments we capture the general commonalities between individuals, reducing the differences between individuals, so the overall fluctuations will be smaller in comparison.

**Table 3:** Accuracy of emotion analysis under subject independence (mean ± std).

**Accuracy**	**Subject_independent**		
	**Arousal(**%**)**	**Valence(**%**)**	**Valence** & **Arousal(**%**)**
Static	63.91 ± 9.9	62.9 ± 9.58	38.98 ± 8.5
Dynamic-10	68.13 ± 9.44	70.73 ± 6.76	44.53 ± 10.43
Dynamic-6	72.97 ± 7.74	74.92 ± 8.92	49.84 ± 8.31
Dynamic-3	78.2 ± 6.74	77.82 ± 8.32	56.17 ± 8.79
Dynamic-1	**91.17** **±4.88**	**90.89** **±3.21**	**74.69** **±7.44**

Bolded values are the best results obtained in different experiments.

**Figure 10:**
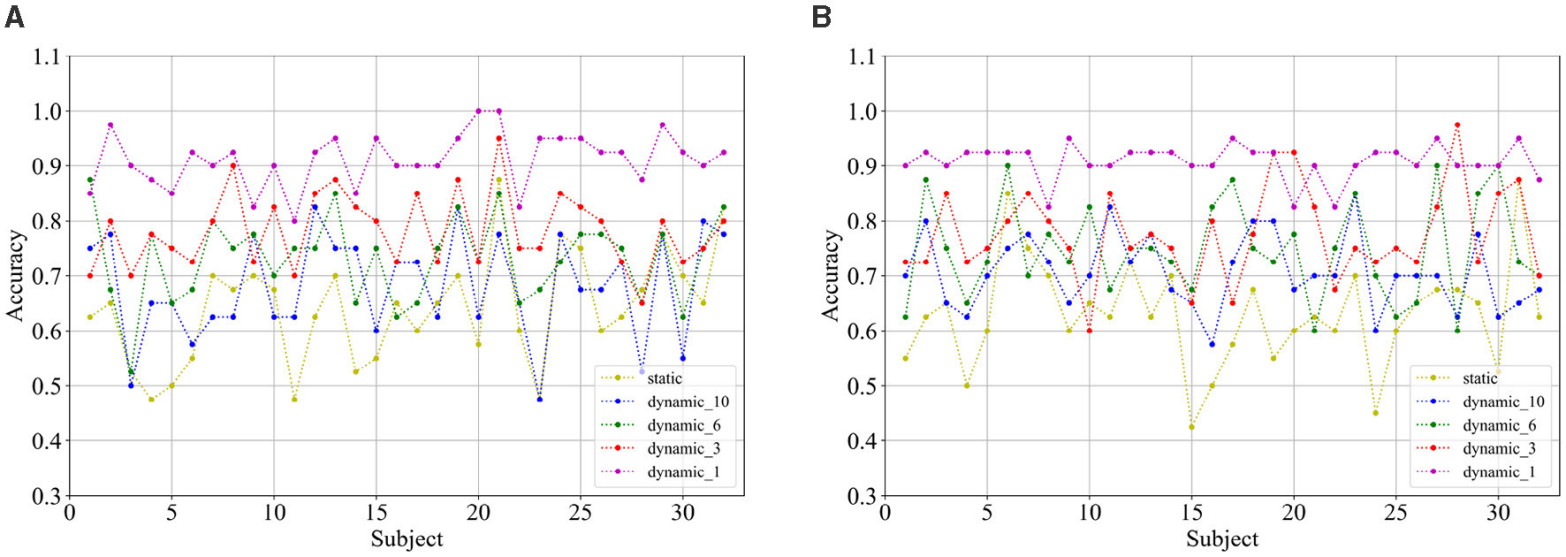
Classification accuracy of **(A)** arousal and **(B)** valence dimensions for each subject under subject independent experiment.

Comparing the categorization under the static brain function network with the different dynamic brain function networks, it can be seen that the categorization performance of the static brain function network is worse than that of the dynamic brain function network. The classification performance of the dynamic brain function network rises gradually with the shortening of the length of the sliding time window, and when the sliding time window is shortened to 1 second, it can be clearly found that there is a significant improvement in the classification accuracy. And the approach achieves a high classification accuracy of 91.17% and 90.89% in the arousal and valence dimensions, 74.69% for the four-class classification, respectively, which fully explains the high temporal resolution of EEG signals, and also indicates that a 1-second sliding time window can relatively adequately capture common feature information across subjects compared to longer sliding time windows, and has an enhanced effect on cross-subject categorization. Figures 10A, B show the classification accuracies of the dynamic and static functional brain networks constructed based on arousal and valence dimensions for subject-independent experiments for each subject.

The Wilcoxon signed-rank test was applied to the results from 32 subjects across three classification scenarios. In the arousal dimension, a significant increase in accuracy was observed as the sliding time window length was shortened, indicating that shorter sliding time windows led to better performance compared to longer ones. In the valence dimension, no significant increase in accuracy was found for “Dynamic-3” compared to “Dynamic-6.” However, in all other cases, there was a significant increase in accuracy when the sliding time window was set to a shorter duration compared to a longer one. For the four-class classification, a similar trend was observed: as the sliding time window was shortened, there was a significant increase in accuracy, demonstrating that shorter sliding time windows were more effective than longer ones.

#### 3.2.3 Subject- and trial-independent experimental results

After the inter-subject LOSO validation is finished, the comparison of the performance of the static brain function network and the different dynamic brain function networks for the cross-subject and -trial LOSO validation experiments continued to be validated, and the results in Table 4 were obtained by averaging the 32 LOSO validations. From the table, it can be found that the classification accuracies of the cross-subject and -trial experiments for binary classification under the static brain function network and different dynamic brain function networks are slightly lower than the results of the cross-subject experiments, and the fluctuation of classification accuracies among the 32 subjects is even larger. The four-classification case witnesses a slightly greater decrease in classification accuracy due to the introduction of more uncertainty across trials, making it more difficult to finely delineate the sentiment.

**Table 4:** Accuracy of emotion analysis under subject and trial independence (mean ± std).

**Accuracy**	**Subject and Trial_independent**		
	**Arousal(**%**)**	**Valence(**%**)**	**Valence** & **Arousal(**%**)**
Static	65.78 ± 11.46	59.06 ± 12.21	37.5 ± 11.66
Dynamic-10	65.63 ± 11.58	68.91 ± 10.81	39.22 ± 12.57
Dynamic-6	68.44 ± 10.42	71.56 ± 10.93	42.34 ± 11.66
Dynamic-3	77.34 ± 8.75	75.47 ± 12.46	51.88 ± 12.42
Dynamic-1	**91.09** **±6.58**	**88.13** **±6.47**	**67.66** **±9.27**

Bolded values are the best results obtained in different experiments.

**Figure 11:**
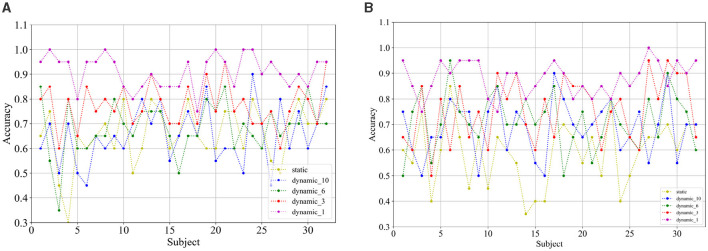
Classification accuracy of **(A)** arousal and **(B)** valence dimensions for each subject under subject and trial independent experiment.

Figures 11A, B show the classification accuracies of the dynamic and static functional brain networks constructed based on arousal and valence dimensions for subject- and trial-independent experiment validation for each subject. However, the trends of classification accuracy for static brain functional networks and dynamic brain functional networks under different sliding time windows are similar to those in the above cross-subject experiments. Both show a gradual increase in classification accuracy as the sliding time window shortens. Moreover, there is a significant increase in accuracy when the sliding time window is 1 second. Along the arousal and valence dimensions, an average classification accuracy of 91.09% and 88.13% is achieved, respectively, and 67.66% in the four-class classification. It can be concluded that dynamic brain functional networks have better performance than static brain functional networks in predicting emotional responses between subjects across trials, and that dynamic brain functional networks under a 1-second sliding time window are more capable of capturing information about the common features among different emotions of different subjects.

The Wilcoxon signed-rank test was conducted on the results from 32 subjects across three classification scenarios. In the arousal dimension, there was no significant improvement in accuracy for “Dynamic-6” over “Dynamic-10” and “Static,” nor for “Dynamic-10” over “Static.” However, in the remaining cases, a significant increase in accuracy was observed when the sliding time window was set to a shorter duration compared to a longer one. In the valence dimension, no significant improvement in accuracy was found for “Dynamic-3” over “Dynamic-6” and “Dynamic-10,” or for “Dynamic-6” over “Dynamic-10.” In the remaining cases, there was a significant increase in accuracy when the sliding time window was set to a shorter duration compared to a longer one. For the four-class classification, significance analysis yielded results consistent with those observed in the arousal dimension, showing a significant increase in accuracy when the sliding time window was set to a shorter duration compared to a longer one, except for the specific comparisons mentioned above.

#### 3.2.4 Comparison with related works

In addition, we compared the results of subject-dependent and subject-independent experiments with the results of other recent works (the other works also used the DEAP data set, and the same subject-independent experiments were conducted with LOSO validation), mainly for subject-independent experiments, as shown in Table 5. As can be seen from Table 5, the subject-dependent experiment achieved the highest accuracy of 99.68% and 100% under both valence and arousal dimensions for our experimental method, which were achieved at sliding time window lengths of 6 seconds and 3 seconds, respectively. We also obtained higher average classification accuracies than most deep learning methods on the valence and arousal dimensions in subject-independent experiments, at 90.89% and 91.17%, respectively. The results of the subject-independent experiments were obtained under a 1-second sliding time window, which fully demonstrates that a sliding time window for fine segmentation of EEG signals is conducive to improving the performance of model generalization across subjects and across subjects and trials.

**Table 5:** Comparison of experimental results with other recent work results.

		**Subject_dependence**		**Subject_independence**	
	**(Features) model/classifier**	**Valence (%)**	**Arousal (%)**	**Valence (%)**	**Arousal (%)**
Liu et al. (2023a)	AP-CapsNet	93.89	95.04	62.71	63.51
Xu G. et al. (2023)	GRU-Conv			67.36	70.07
Fan et al. (2024)	LResCapsule	97.45 ± 1.49	97.58 ± 1.31	61.52	66.84
Hou et al. (2024)	MECAM	96.95 ± 2.67	96.59 ± 2.30	**94.76** **±3.77**	**93.44** **±4.06**
Tang et al. (2024)	MD^2^GRL	96.51 ± 2.89	95.77 ± 2.35	92.58 ± 3.80	92.04 ± 3.96
Han et al. (2024)	(DE) MS-ERM	90.45	91.31	85.40	86.66
Fan et al. (2024)	LResCapsule	97.45 ± 1.49	97.58 ± 1.31	61.52	66.84
Ours	(MI_Cp,Eloc,Eg) SVM	**99.69** **±1.74**	**100** **±0**	90.89 ± 3.21	91.17 ± 4.88

Bolded values are the best results obtained in different experiments.

## 4 Discussion

### 4.1 Emotion recognition results in different functional brain regions

**Figure 12:**
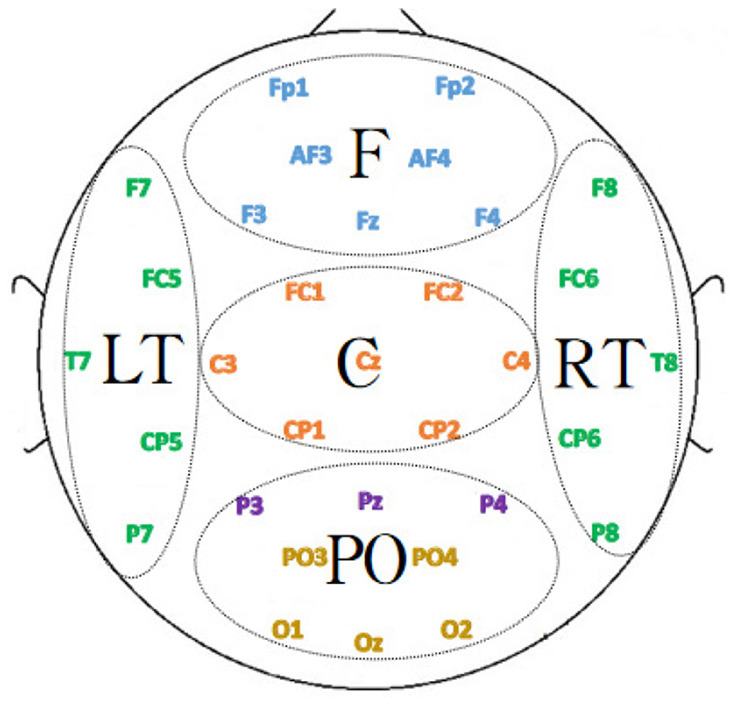
Functional division of the brain.

The DEAP dataset studied in this paper was acquired with a 32-electrode EEG device. These 32 electrodes are divided into the following five regions: frontal, temporal, occipital, parietal and central, and the electrodes in different regions were represented by five different colors in Figure 12.

To investigate the contribution of each brain region to emotion classification, the electrodes were divided into different regions according to the position, and the region-related dynamic brain function network was constructed for analysis. Since the number of EEG channels in the parietal region was relatively small, the EEG channels in the parietal and occipital regions were combined. Additionally, due to the regional separation of the left and right temporal lobes, the left and right temporal lobes were selected to construct the dynamic brain function network separately for analysis. The specific division is depicted in Figure 12 where the 32 EEG channels were divided into five regions, F, LT, RT, C, and PO, corresponding to the frontal, left temporal, right temporal, central, occipital, and parietal lobes. The dynamic brain function network of each region with a sliding time window length of 1 was constructed by the previous process. The features of the network attributes (local efficiency, global efficiency, and clustering coefficient) were extracted and put into the SVM for classification.

#### 4.1.1 Subject-dependent experimental results

The comparison of the emotion recognition accuracies of the dynamic brain function networks in each brain region under a subject-dependent experimental setup is shown in Table 6. For the arousal dimension, the PO region achieved the highest average accuracy of 86.25% among the brain region classifications. The occipital lobe processes visual information that elicits emotion, and the parietal lobe can help individuals assess the intensity of emotion by integrating various sensory information from inside and outside the body. Therefore, the parietal and occipital regions are able to integrate visual and bodily sensory information and play a dominant role in judging emotional arousal, leading to more accurate judgments. For the valence dimension, the RT region achieved the highest mean classification accuracy of 83.44%, and the LT region achieved a result of 83.13%. Compared to the average classification accuracy of each brain region in the arousal dimension, which did not differ much, the results of the remaining three regions (F, C, and PO) in the valence dimension differed more from those of the RT and LT regions. Individuals' judgments of emotional valence are often based on the in-depth processing and understanding of information, which involves the processing of past memories. For the reason that the medial part of the temporal lobe is closely related to memories, the left and right temporal lobes achieved better results than the other brain regions, which reveals that the left and right temporal lobes dominate the personal processing of emotional pleasure levels.

**Table 6:** Subject-dependent emotion recognition results across brain regions (mean ± std).

**Accuracy**	**Subject_dependent**	
	**Arousal(**%**)**	**Valence(**%**)**
F	80.63 ± 23.84	69.38 ± 24.23
LT	80.31 ± 28.99	83.13 ± 24.42
RT	83.75 ± 24.84	**83.44** **±23.8**
C	82.5 ± 22.91	70.63 ± 29.47
PO	**86.25** **±20.12**	69.69 ± 24.56

Bolded values are the best results obtained in different experiments.

#### 4.1.2 Subject-independent experimental results

**Figure 13:**
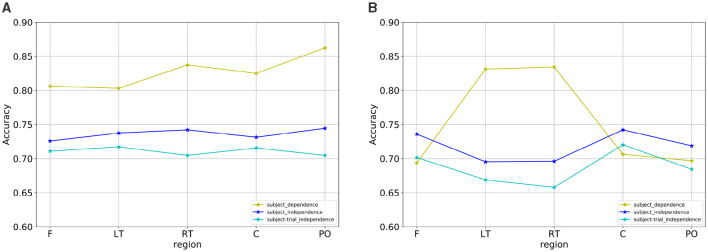
Emotion recognition accuracy across brain regions for **(A)** arousal and **(B)** valence dimensions.

Under the subject-independent experimental setup, the emotion recognition accuracy of the dynamic brain function network in each brain region is compared, as shown in Table 7. For the arousal dimension, the classification results of each brain region were lower than those under the subject-dependent experimental setup, among which the PO region still achieved the highest average classification accuracy of 74.45%. Figure 13 depicts the emotion recognition accuracy across brain regions for arousal and valence dimensions. As can be seen in Figure 13A, the classification results of the remaining brain regions were also similar to the trend of changes in the average classification accuracies of the different brain regions obtained under the subject-dependent experimental setup. For the valence dimension, the classification results of each brain region were lower than those obtained in the subject-dependent experimental setup. By observing the trend of the average classification accuracy of different brain regions in Figure 13B, it can be seen that the trend of the classification results of each brain region under the subject-independent setup is essentially the opposite of that obtained under the subject-dependent setup. Region C achieved the best average classification accuracy of 74.22%; region F also achieved a classification result of 73.59%; and the left and right temporal lobes, which had better results in the subject-dependent setup, achieved the relatively worst results. There is a fundamental difference between the subject-independent and subject-dependent settings in the way they deal with individual variability. In the subject-dependent setting, the model is trained separately for each subject, i.e., part of a given subject's data is used for training and the rest of the data is used for testing, thus capturing the emotion-related brain activity patterns for each subject specifically. In the subject-independent setting, the model is based on the training of certain subjects and is used to predict certain untrained subjects, so the model needs to capture the emotion-related brain activity patterns that are common to different subjects. The experimental results demonstrate that the central and frontal regions mainly process basic shared information about emotions, while other regions are influenced by the subjective feelings of the subjects and focus more on emotionally personalized information, which is why the central and frontal regions performed better in the subject-independent experiments.

**Table 7:** Subject-independent emotion recognition results across brain regions (mean ± std).

**Accuracy**	**Subject_independent**	
	**Arousal(**%**)**	**Valence(**%**)**
F	72.58 ± 6.72	73.59 ± 7.65
LT	73.75 ± 7.76	69.53 ± 7.97
RT	74.22 ± 7.84	69.61 ± 6.71
C	73.13 ± 5.12	**74.22** **±7.17**
PO	**74.45** **±7.41**	71.88 ± 6.93

Bolded values are the best results obtained in different experiments.

#### 4.1.3 Subject- and trial-independent experimental results

The comparison of emotion recognition accuracy of the dynamic brain function network in each brain region under the subject- and trial-independent experimental setup is shown in Table 8. For each brain region, as can be seen in Figures 13A, B, the average classification accuracy results for each brain region in the arousal and valence dimensions were lower than the experimental results in the subject-independent setting, and the trends were similar between regions. The average classification results obtained in each brain region in the arousal dimension did not differ much, with the best result of 71.72% obtained in the LT region. In the valence dimension, the best classification accuracy result of 72.03% was obtained in the central region, in line with the results obtained in the subject-independent setting.

**Table 8:** Subject- and trial-independent emotion recognition results across brain regions (mean ± std).

**Accuracy**	**Subject and Trial_independent**	
	**Arousal(**%**)**	**Valence(**%**)**
F	71.09 ± 12.79	70.16 ± 10.34
LT	**71.72** **±11.96**	66.88 ± 8.55
RT	70.47 ± 11.55	65.78 ± 8.67
C	71.56 ± 11.89	**72.03** **±11.78**
PO	70.47 ± 10.41	68.44 ± 10.78

Bolded values are the best results obtained in different experiments.

### 4.2 Experimental results of setting different thresholds

Threshold settings during the construction of the whole-brain functional network were explored. In addition to setting the data at position 0.3 of the ascending numerical order as the threshold, the data at positions 0.5 and 0.7 are also set as the threshold, and the classification results of each dynamic and static brain functional network are shown in Tables 9, 10 for arousal and valence dimension. When the data at position 0.5 or 0.7 is set as the threshold, the changing trends of classification accuracies of different static and dynamic brain functional networks under each sliding time window are approximately consistent with the changing trend when the data at position 0.3 is as the threshold. That is, better performance is achieved when the window division accuracy is smaller. In the arousal dimension, t = 1,3,6 and static cases, and in the valence dimension, t = 1,6,10 cases, the best results are achieved with 0.3 positional data as the threshold, and in other cases, better results are achieved with 0.5 or 0.7 positional data as the threshold. In this regard, by observing the elemental composition of the correlation matrix before thresholding, it can be found that when using MI to calculate the correlation after data processing, the change in the correlation value between channels was within a small range. Therefore, the sparsity of connectivity in the functional brain networks after setting different thresholds might be similar, and the obtained results were also similar.

**Table 9:** Classification results under different thresholds/arousal (mean ± std).

**Accuracy**	**Subject_independent/arousal**				
	**t=1(**%**)**	**t=3(**%**)**	**t=6(**%**)**	**t=10(**%**)**	**Static(**%**)**
0.3	**91.17** **±4.88**	**78.2** **±6.74**	**72.97** **±7.74**	68.13 ± 9.44	**63.91** **±9.9**
0.5	88.91 ± 5.96	76.95 ± 6.75	72.27 ± 6.26	68.91 ± 7.39	63.05 ± 10.49
0.7	86.88 ± 7.04	75.94 ± 7.15	70.86 ± 7.77	**71.02** **±7.6**	62.97 ± 8.83

Bolded values are the best results obtained in different experiments.

**Table 10:** Classification results under different thresholds/valence (mean ± std).

**Accuracy**	**Subject_independent /valence**				
	**t=1(**%**)**	**t=3(**%**)**	**t=6(**%**)**	**t=10(**%**)**	**Static(**%**)**
0.3	**90.78** **±3.21**	77.58 ± 8.3	**74.77** **±8.82**	**70.63** **±6.67**	62.89 ± 9.58
0.5	86.88 ± 7.04	77.34 ± 6.7	72.19 ± 7.26	68.52 ± 7.95	**63.91** **±9.44**
0.7	90 ± 6.73	**77.81** **±8.4**	72.66 ± 7.21	69.06 ± 6.84	62.27 ± 7.38

Bolded values are the best results obtained in different experiments.

### 4.3 Experimental results for differences between frequency bands

In subject-independent experiments, we generated functional brain network graph features using interband differences under a 1-second sliding time window to assess the contribution of functional brain networks with different interband differences to emotion classification (alpha_theta denotes the inter-amplitude and inter-phase difference under the alpha and theta bands). The average classification accuracies of different inter-band difference features are shown in Table 11. For classification based on amplitude and phase differences between the bands, in the arousal dimension, gamma_beta achieves the best classification performance, followed by gamma_theta, and beta_alpha has the worst result. In the valence dimension, beta_theta achieves the best performance, followed by beta_alpha, and alpha_theta has the worst result. However, the classification results for the difference between the bands were worse than those for all the bands combined. This is similar to the results of numerous research experiments (Xu G. et al., 2023; Lin et al., 2023; Hou et al., 2022; Li C. et al., 2023) conducted by dividing the frequency bands, where the classification accuracy of all bands is higher than that of a single band, indicating that there is an effect of information compensation between the bands.

**Table 11:** Classification results for inter-band differences (mean ± std).

**Accuracy**	**Subject_independent / t=1**	
	**Arousal(**%**)**	**Valence(**%**)**
alpha_theta	79.45 ± 6.02	77.81 ± 6.4
beta_alpha	77.89 ± 7.1	79.22 ± 6.92
beta_theta	79.14 ± 6.48	79.45 ± 5.92
gamma_alpha	79.3 ± 5.97	78.98 ± 6.28
gamma_beta	81.25 ± 6.47	78.98 ± 6.34
gamma_theta	79.84 ± 4.8	78.36 ± 6.84
all	**91.17** **±4.88**	**90.89** **±3.21**

Bolded values are the best results obtained in different experiments.

### 4.4 Applied to the SEED dataset

To verify the effectiveness of our approach, we also apply our experimental methodology to another commonly used dataset which is the SEED dataset (Zheng and Lu, 2015). The SEED dataset recorded EEG signals from 15 subjects (7 males and 8 females with an average age of 23.27 years old) watching 15 segmented videos (including positive, neutral, and negative types, 3–5 min per video segment), with three repetitive acquisitions at intervals for each subject. The EEG sampling frequency was 1,000 Hz and there were 62 EEG acquisition channels. The dataset has preprocessed signals provided downsampled to 200 Hz and artifacts removed from the signals using a 0–75 Hz bandpass filter. The preprocessed signal is selected for validation experiments in this paper. In order to benchmark the SEED dataset against the DEAP dataset, a standardization was chosen to retain the 60 seconds of data from 90 seconds to 150 seconds of each segment and to combine the EEG signals from the 3 acquisitions for each subject, so that the data size for each subject was 45 trials * 62 channels * 12,000 (200 Hz*60s).

The SEED dataset was sequentially processed similarly to the DEAP dataset described above, with wavelet analysis to delineate frequency bands, construction of dynamic brain function networks and extraction of network attribute features, feature selection and classification. The three kinds of experiments mentioned above were done on the processed data separately: subject-dependent, subject-independent, and subject- and trial-independent experiments. Here we only selected sliding time windows of 6, 3, and 1 seconds to construct dynamic brain function networks. Table 12 lists the results under various experiments. The best results of 95.56% were obtained under the subject-dependent experiment with a 3-second sliding window, and another 80% and 95% were obtained under the 1-second and 6-second sliding windows, respectively. Under the subject independent experiment, the best results were obtained 97.04% under the 1-second sliding window, in addition to 81.63% and 73.19% under the 3- and 6-second sliding windows, respectively. The best results 93.94% were achieved under subject- and trial- independent experiment under 1 second sliding time window and additionally 76.06% and 71.82% were achieved under 3 and 6 second sliding time windows. From the above results, it can be seen that the trend of the results from different sliding time windows is similar to that of the DEAP dataset, and the method proposed in this paper also performs well on the SEED dataset.

**Table 12:** Three classification on the SEED dataset (mean ± std).

**Accuracy**	**subject_dependent(*%*)**	**subject_independent(*%*)**	**subject and trial_independent(*%*)**
Dynamic-6	95 ± 16.61	73.19 ± 6.1	71.82 ± 8.66
Dynamic-3	**95.56** **±16.63**	81.63 ± 4.55	76.06 ± 8.04
Dynamic-1	80 ± 28.84	**97.04** **±2.65**	**93.94** **±4.29**

Bolded values are the best results obtained in different experiments.

### 4.5 Limitations

While this study presents competitive results in EEG-based emotion recognition using dynamic brain functional networks, it also has several limitations that could affect the generalizability of the findings: (1) During the experiment, we encountered a limitation where the code running time increased as the sliding time window length was shortened. This trade-off between temporal resolution and computational efficiency poses a significant challenge for future research. It is crucial to develop effective methods to reduce the time complexity of EEG signal analysis while preserving the high temporal and spatial resolution necessary for accurate emotion recognition. (2) In the study of different brain regions, only the perspective of individual brain regions is investigated. However, in reality, each brain region does not exist in isolation, and there are complex network connections and interactions among them. To conduct a more comprehensive and nuanced biological interpretation of the brain regions requires collaboration with researchers in the field of neuroscience. (3) Additionally, the differences in acquisition equipment, emotion-evoking stimuli, and emotion labeling methods among different datasets may pose challenges to the analysis and processing of EEG signals. In the future, we plan to validate our method on different datasets, aiming to obtain more realistic and general experimental results.

## 5 Conclusion

In this paper, we focus on feature extraction for EEG emotion recognition and propose a novel approach by fusing feature information from EEG time-frequency space using the dynamic brain functional network method. We extensively compare the performance of static and dynamic brain functional networks under various sliding time windows for emotion recognition. Our findings indicate that the dynamic brain functional network outperforms the static brain functional network in capturing more relevant information, thus enhancing emotion categorization. This advancement holds significant potential for the fields of artificial intelligence and emotion recognition. In the future, we can monitor emotional changes dynamically and discover more detailed emotional responses, which can be applied to, for example, psychological disease detection, driver emotion monitoring, and emotional companion robots. These applications can sense the emotional changes of patients with psychological diseases in a timely manner while providing them with correct feedback and treatment, remind drivers to adjust their emotions promptly to reduce the occurrence of road accidents, and enable emotional companion robots to sense the emotional changes of humans in a timely manner while providing appropriate emotional support, among other things.

## Data Availability

The original contributions presented in the study are included in the article/supplementary material, further inquiries can be directed to the corresponding authors.
